# G2-S16 dendrimer microbicide does not interfere with the vaginal immune system

**DOI:** 10.1186/s12951-019-0496-9

**Published:** 2019-05-15

**Authors:** Alba Martín-Moreno, Daniel Sepúlveda-Crespo, Mª Jesús Serramía-Lobera, Ana Judith Perisé-Barrios, Mª Angeles Muñoz-Fernández

**Affiliations:** 1 Sección Inmunología, Laboratorio InmunoBiología Molecular, Hospital General Universitario Gregorio Marañón (HGUGM), Instituto de Investigación Sanitaria Gregorio Marañón (IiSGM), and Spanish HIV-HGM BioBank, Madrid, Spain; 2 Networking Research Center on Bioengineering, Biomaterials and Nanomedicine (CIBER-BBN), Madrid, Spain

**Keywords:** G2-S16 dendrimer, Microbicide, Immunity, Antigen presenting cells, Lymphocytes

## Abstract

It is essential that prophylactic drugs do not interfere with the normal function of the immune system. The use of nanoparticles as vaginal microbicides is a promising prevention strategy against sexually transmitted infections. With that aim, our group is working with the G2-S16, a second generation carbosilane dendrimer with sulfonate groups in the periphery, which has been previously shown to be effective against HIV-1 and HSV-2 infection, and it is now on the road to clinical trials. Our objective in this new study is to assess the effects of G2-S16 on the immune barrier of the female reproductive tract. The expression of differentiation, maturation and activation markers was measured in epithelial cells, dendritic cells, M and GM macrophages, and T cells using RT-qPCR and flow cytometry. The results demonstrate that G2-S16 does not alter the natural immunity of the vagina, strongly supporting the biosafety of this dendrimer for clinical use.
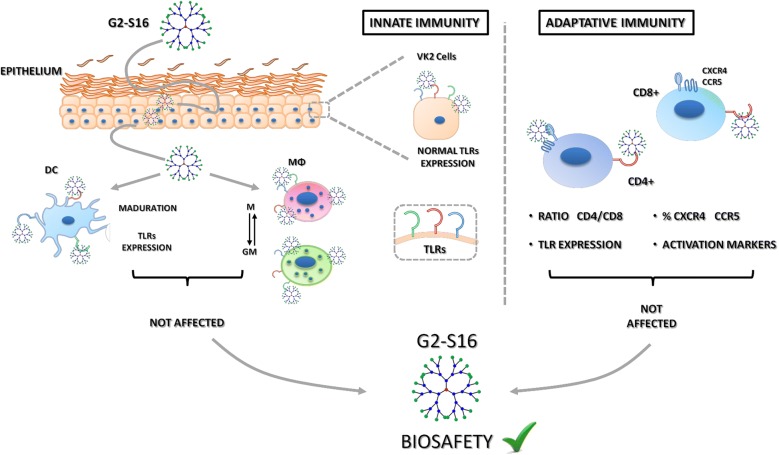

## Background

Vaginal microbicides offer a promising strategy in preventing sexual transmission of human immunodeficiency virus type 1 (HIV-1) and other genital tract infections [1, 2]. The microbicides should not interfere in the ability of genital mucosa to provide protection against other pathogens. The cervicovaginal epithelial cells, dendritic cells (DCs), tissue-resident macrophages (MØs), and T-lymphocytes associated with the female reproductive tract (FRT) play a crucial role in the early recognition of pathogens and launching of an immune response against vaginal infections [3, 4].

The innate immune system constitutes the first line of defense against viral infection, especially after disruption of mucosal epithelium that occurs during sexual intercourse. The innate immune response leads to inflammation, recruitment of immune cells from the bloodstream to tissue, and activation of adaptive immunity to specifically fight the infection [5]. Activation of the immune system is triggered when pathogen-associated molecular patters are recognized by pattern recognition molecules like Toll-like receptors (TLRs) on the host cells, remarkably epithelial cells, mucosal DCs, tissue MØs and mucosal resident T-cells [6]. As a result of TLRs stimulation, an inflammatory response is initiated after activation of several intracellular signaling pathways and the subsequent production of pro-inflammatory cytokines [7, 8]. Ten different TLRs (TLR1–TLR10) have been identified in humans [9, 10], and each one of them specifically recognizes different antigens [11].

Dendritic cells are cells of the innate immune system, but their role as antigen presenting cells (APCs) makes them a link between the innate and the adaptive immune system. DCs are sentinels that capture and process antigens, mature and migrate to secondary lymphoid tissues where they present the antigens and activate T-cells.

Also working as APCs are the MØs. MØs secrete cytokines that recruit the rest of the immune cells to sites of infection [12], and orchestrate the immune response. MØs are classified as pro-inflammatory [M1 or GM-CSF-polarized MØs (GM-MØs)] or anti-inflammatory [M2 or M-CSF-polarized MØs (M-MØs)]. GM-MØs are phagocytic cells that eliminate pathogens, infected or cancerous cells, or cell debris, and clean the tissues, while secreting pro-inflammatory cytokines and thus contributing to tissue destruction. On the other hand, M-MØs activate T-cell system, secrete anti-inflammatory cytokines and promote tissue regeneration. Hence, a correct balance of GM/M-MØs maintains homeostasis, whereas disequilibrium can induce chronic inflammation and disease [13].

**Fig. 1:**
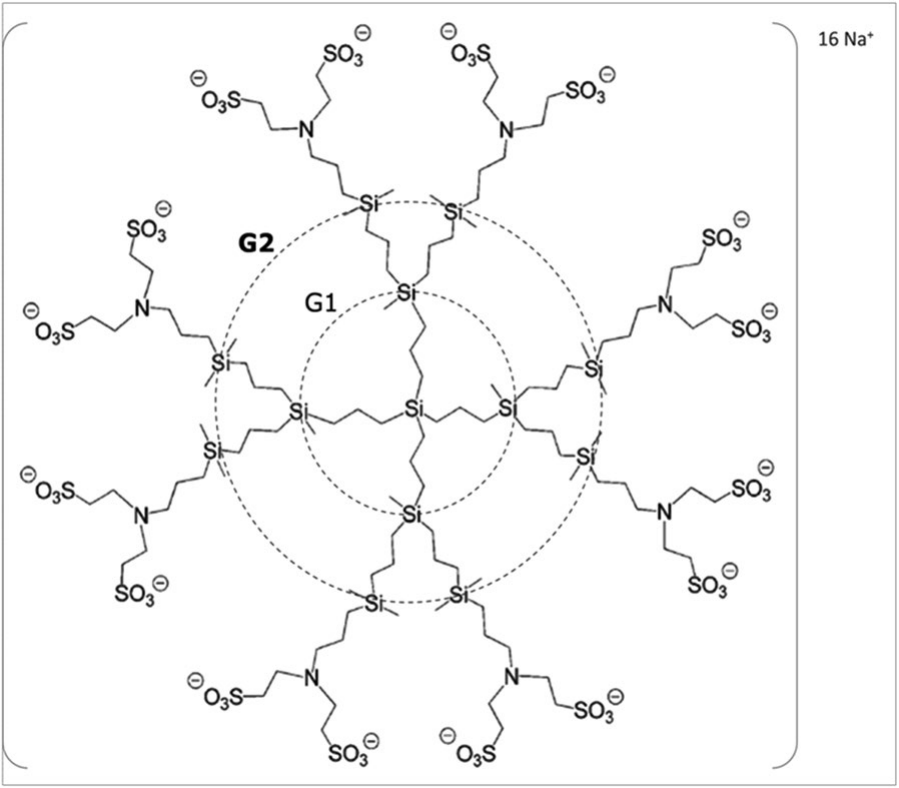
Chemical structure of second-generation polyanionic carbosilane dendrimer G2-S16. The generation were defined as the number of repeated layers of silicon atoms forming the dendrimer. The capping layer consists of 16 sulfonate groups (–SO3–). The molecular formula is C112H244N8Na16O48S16Si13 and the molecular weight is 3717.15 g/mol

It is necessary for any microbicide not only to prevent infection but also not to interfere with the function of the immune cells. Nanotechnology is being widely used in the design of novel systems as microbicides capable of disrupting HIV transmission [14, 15]. Polyanionic carbosilane dendrimer G2-S16 has emerged as a promising potential microbicide. G2-S16 (see Fig. 1 for details about the dendrimer) belongs to a heterogeneous group of compounds acting as HIV/herpes simplex virus-type 2 (HSV-2) entry inhibitors that have shown to be harmless and effective in different in vitro and in vivo models [16–20]. G2-S16 is currently on the road to clinical trials, but in order to further guarantee the safety of its use as vaginal gel, we here tested the effect of this dendrimer on cell viability, TLR expression and differentiation, maturation and activation of the main immune cells localized in the vaginal mucosa. Our results clearly show that G2-S16 does not interfere with the normal function of the mucosal barrier, and vaginal innate or adaptive immunity.

## Results

### Cytotoxicity of G2-S16 dendrimer on dendritic cells and monocyte-derived macrophages

**Fig. 2:**
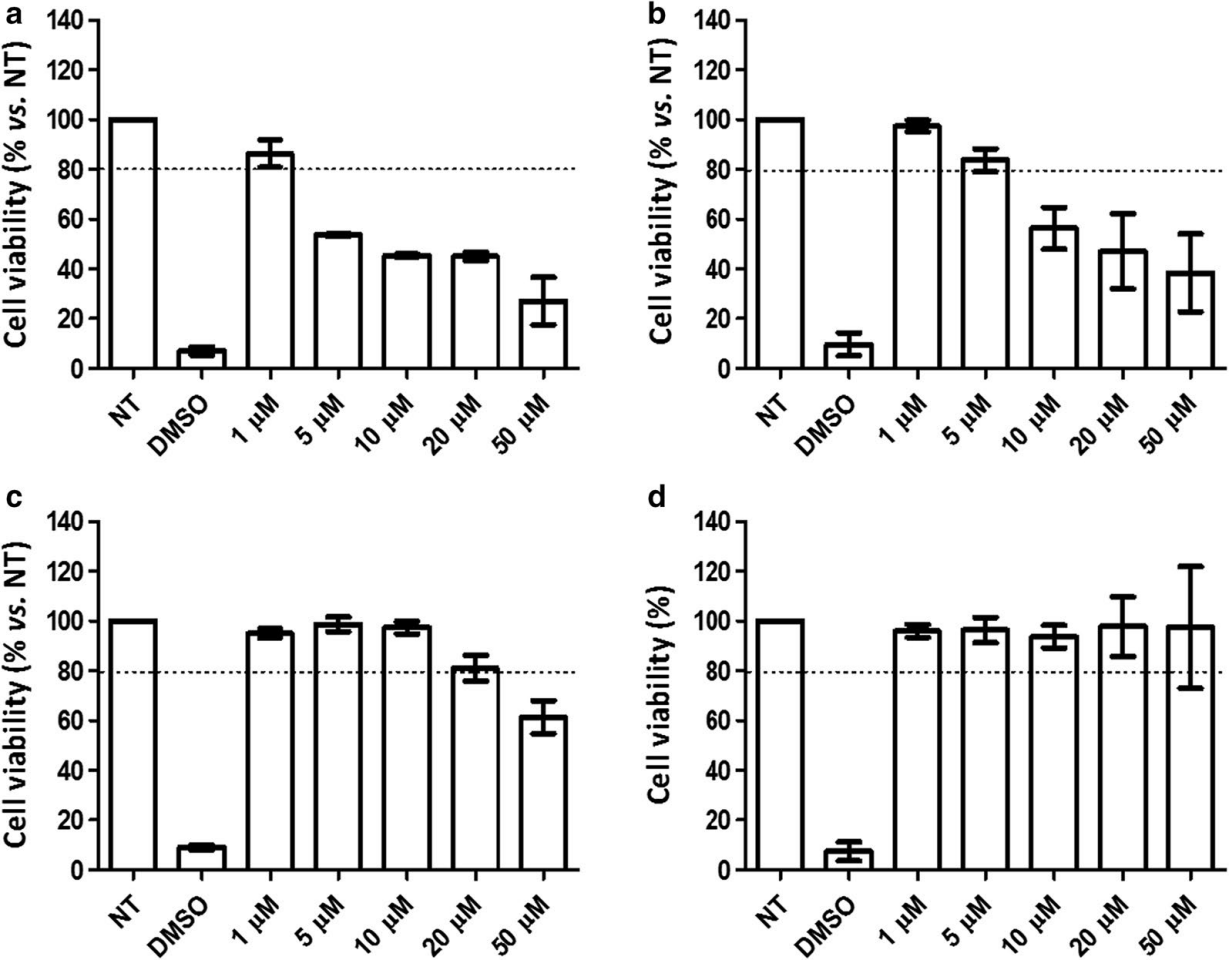
Cytotoxicity associated to G2-S16 in dendritic cells and macrophages. **a** iDCs, **b** mDCs, **c** GM-MØs or **d** M-MØs were loaded with increased concentrations of G2-S16 (ranged from 1 to 50 µM) or treated with 20% of DMSO (control of cell death) for 48 h. The percent of cell viability was calculated by MTT as optical density of treated condition/non-treated control (NT) × 100. The 80% of viability was set as limit of toxicity. Data are represented as mean ± SD of three experiments performed in triplicate. DMSO: dymethyl sulfoxide; iDCs: immature dendritic cells; mDCs: mature dendritic cells; GM-MØs: pro-inflammatory or M1 macrophages; M-MØs: anti-inflammatory or M2 macrophages

We selected 10 µM as the in vitro working concentration for G2-S16 in epithelial VK2/E6E7 cells, peripheral blood mononuclear cells (PBMCs) and purified T-cells as determined in previous published studies [19, 21]. However, the cytotoxicity of G2-S16 on DCs or MØs had not been evaluated. Thus, DCs and MØs were treated for 48 h with increasing concentrations of G2-S16 dendrimer (1–50 μM) that were considered toxic when the survival rate was < 80%. DMEM medium and 20% dimethyl sulfoxide (DMSO; Sigma) were used as non-treated and cell death controls, respectively. G2-S16 was non-toxic at a concentration of 1 µM on iDCs (Fig. 2a) and of 5 µM on mDCs (Fig. 2b) whereas was considered non-toxic up to 10 µM on GM-MØs (Fig. 2c). G2-S16 was non-toxic over the full range of concentrations assayed on M-MØs (up to 50 µM; Fig. 2d). Thus, the selected non-toxic working concentrations for the following in vitro assays were of 1 µM in DCs and 10 µM in MØs.

### Toll-like receptor expression in vaginal epithelial cells remains unaffected in the presence of G2-S16 dendrimer

**Fig. 3:**
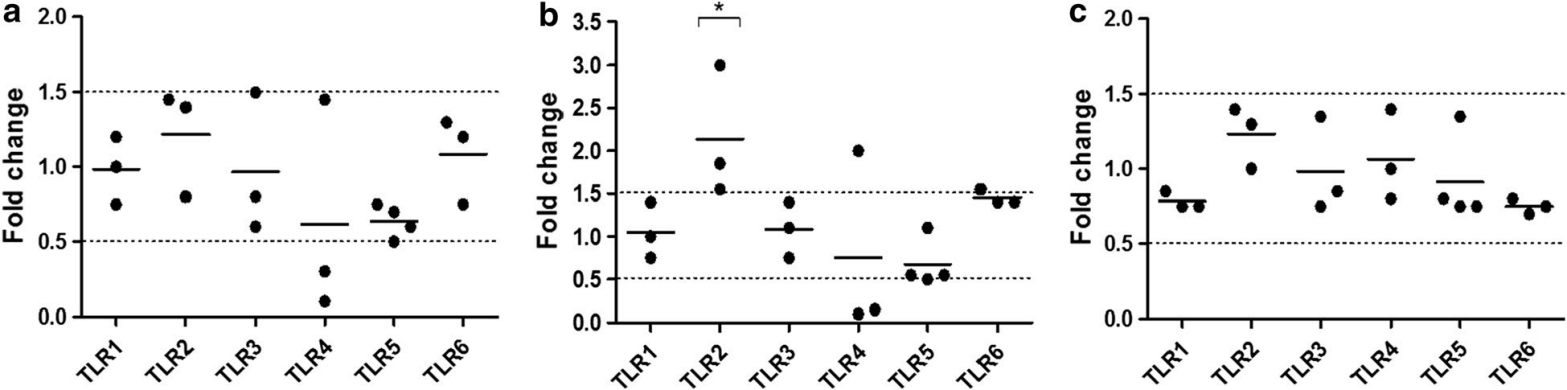
Quantification of TLR gene expression on treated VK2/E6E7. TLR1–10 mRNA expression was determined by RT-qPCR in VK2/E6E7 cells treated with G2-S16 (10 µM) for **a** 3 h, **b** 6 h and **c** 18 h. Insufficient detection of certain TLR mRNA expression levels by RT-PCR was not included here to simplify the representation. Data shows relative mRNA levels; analyzed gene was normalized to TATA box binding protein expression (TBP) and referred to non-treated cells. Individual values (dots) and mean (bar) of at least three independent experiments are shown. Dashed lines indicate threshold values

The epithelium is the first line of defense against potential pathogens or harming substances, and, although frequently underestimated, it is a key component of the immune system. It has been observed that activation of TLRs in the epithelial cells induces inflammatory responses and the disruption of tight junctions, increasing mucosal permeability and facilitating microbe infiltration [22]. In order to test the safety of G2-S16 as vaginally-applied microbicide, we used TaqmanRT-qPCR to evaluated the change in expression of TLR1–10 in human vaginal epithelial cell line VK2/E6E7 exposed to G2-S16 (10 µM) versus non-treated VK2/E6E7 cells for short (3 h), medium (6 h) or long (18 h) times. cDNA was amplified using the specific PCR forward and reverse primer pairs described in Table 1. A peak in TLR2 expression was observed at 6 h (*p *< 0.05), but the expression was back to basal levels by the 18 h time-point (Fig. 3). Exposure to G2-S16 did not modify the expression of any other TLR from TLR1 to TLR6 in VK2/E6E7 cells. mRNA expression of TLR7–10 in VK2/E6E7 cells was undetectable by RT-PCR. These results strongly suggest that the immune function of the vaginal epithelium is not affected after exposure of our G2-S16 dendrimer.

**Table 1:** Real time PCR primer pairs used for quantification of human TLR mRNA

TLR	Forward primer	Reverse primer	Amplicon size (bp)
LR1	GGTCTTGCTGGTCTTAGGAGAGAC	CTGAAGTCCAGCTGACCCTGTAGCTTCACG	372
TLR2	GGCCAGCAAATTACCTGTGTG	CTGAGCCTCGTCCATGGGCCACTCC	637
TLR3	CGGGCCAGCTTTCAGGAACCTG	GGCATGAATTATATATGCTGC	400
TLR4	TGCAATGGATCAAGGACCAGAGGC	GTGCTGGGACACCACAACAATCACC	449
TLR5	CCTCATGACCATCCTCACAGTCAC	GGCTTCAAGGCACCAGCCATCTC	355
TLR6	CCAAGTGAACATATCAGTTAATACTTTAGGGTGC	CTCAGAAAACACGGTGTACAAAGCTG	358
TLR7	CTCCCTGGATCTGTACACCTGTGAG	CTCCCACAGAGCCTTTTCCGGAGCT	551
TLR8	GTCCTGGGGATCAAAGAGGGAAGAG	CTCTTACAGATCCGCTGCCGTAGCC	581
TLR9	GCGAGATGAGGATGCCCTGCCCTACG	TTCGGCCGTGGGTCCCTGGCAGAAG	510
TLR10	CAGAGGTCATGATGGTTGGATGG	GACCTAGCATCCTGAGATACCAGGGCAG	256

### G2-S16 dendrimer does not alter expression of TLR in monocyte-derived macrophages, but increases expression of TLR2 and TLR7 in iDCs

**Fig. 4:**
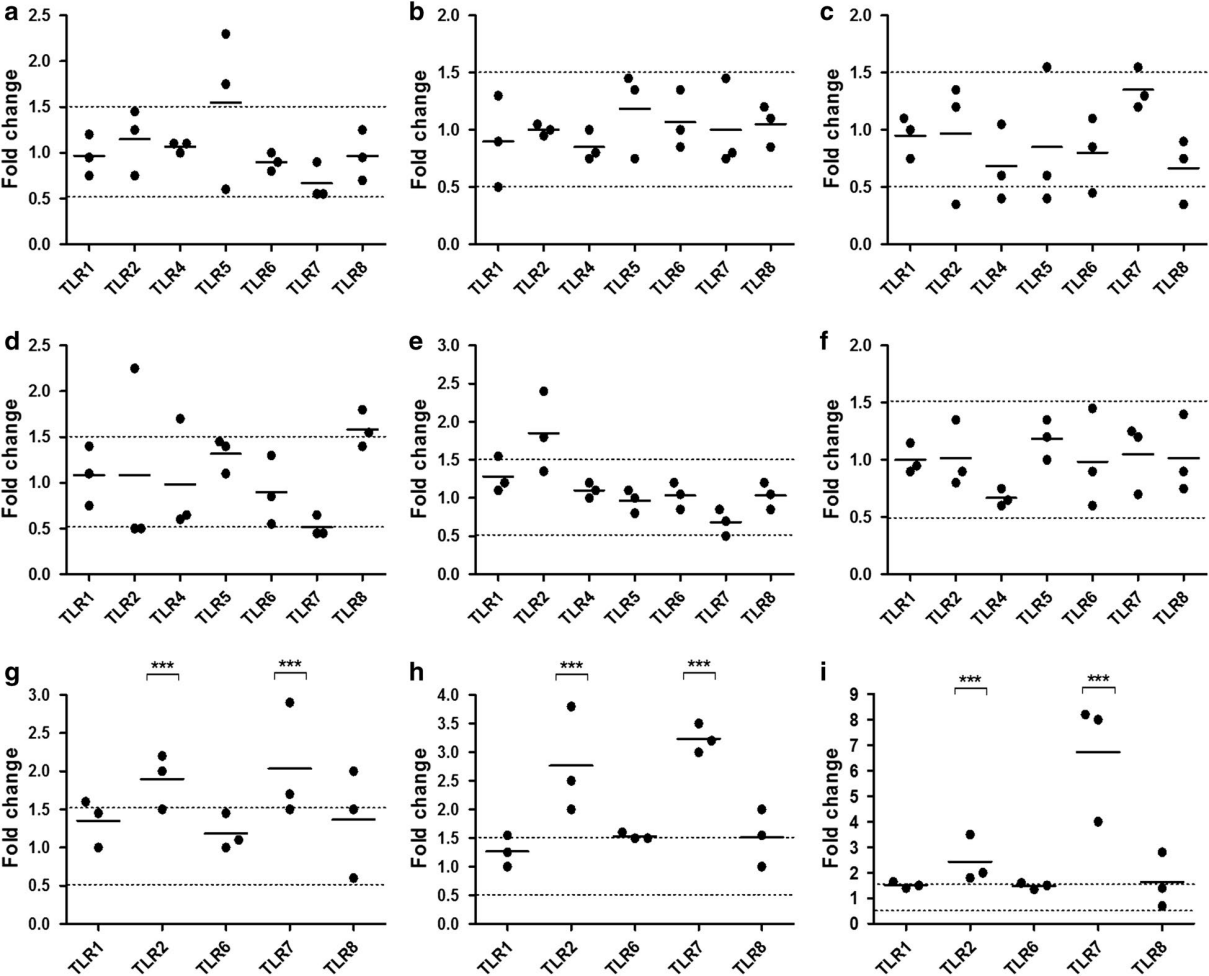
Quantification of gene expression on treated iDCs M-MΦs and GM-MΦs. TLR1–10 mRNA expression was determined by RT-qPCR in **a**–**c** M-macrophages (M-MΦs), **d**–**f** GM-macrophages (GM-MΦs) or **g**–**i** immature dendritic cells (iDCs) treated with G2-S16 (1 µM for iDCs and 10 µM for M- and GM-MΦs) for **a**, **d**, **g** 3 h, **b**, **e**, **h** 6 h and **c**, **f**, **i** 18 h. Insufficient detection of certain TLR mRNA expression levels by RT-PCR was not included here to simplify the representation. Data shows relative mRNA levels; analyzed gene was normalized to TATA box binding protein expression (TBP) and referred to non-treated cells. Individual values (dots) and mean (bar) of at least three independent experiments are shown. Dashed lines indicate threshold values

The next line of defense behind the vaginal epithelium is formed by tissue-resident cells of the innate immune system, especially DCs and MØs. Both cells types recognize pathogens through TLRs, whose activation launches a signaling cascade that derives in expression of pro-inflammatory cytokines, cell maturation and proliferation, and consequent activation of the immune response. To examine the modulation of the TLR expression in these cell types by G2-S16, we measured the mRNA expression of TLR1–10 in iDCs, and monocyte-derived M-MØs and GM-MØs after treatment with G2-S16 (at 1 µM and 10 µM, respectively) for short (3 h), medium (6 h) or long (18 h) times. Expression of TLR3–5, 9 and 10 in iDCs, and TLR3, 9 and 10 in M-MØs and GM-MØs was under the detection limits of the RT-qPCR. In M-MØs and GM-MØs, no significant differences were observed in TLR1,2,4–8 expression after treatment with G2-S16 (Fig. 4a–f). This suggests that G2-S16 dendrimer does not cause a basal activation or alteration in MØ function.

Interestingly, G2-S16 significantly up-regulated TLR2 (2–3-fold; *p *< 0.001) and TLR7 (2–7-fold; *p *< 0.001) at all studied times (Fig. 4g–i). Previous studies showed that TLR2 engagement depends on its expression [23], and leads to DC activation [24], and production and release of IL-12 [25]. Similarly, TLR7 initiates a signaling cascade leading to DC maturation, and in the case of plasmacitoid DCs, it causes a release of type-I IFN. Our results suggested that G2-S16 dendrimer could be causing DC activation, leading to the following assays to make sure that the increase in TLR expression caused by the G2-S16, does not cause an unwanted DC maturation.

### G2-S16 does not affect the maturation of dendritic cells

DCs are antigen-presenting cells that play a crucial role during the initiation and regulation of both innate and adaptive immunity. iDCs reside in peripheral tissues and are sentinels that capture antigen particles via TLR receptor, which leads to their activation. Upon activation, iDCs undergo maturation and migrate towards the draining lymph node, where mDCs expressing increased levels of co-stimulatory molecules, produce cytokines and interact with naïve T-cell, initiating primary T-cell-mediated immune responses [26]. Therefore, the observed over-expression of TLR2 and TLR7 induced by exposure to G2-S16 could influence their phenotype and their ability to mature, thus impairing their function, or cause a basal DC maturation and consequent chronic inflammation. We examined the maturation of the DCs after G2-S16 dendrimer exposure in vitro.

**Fig. 5:**
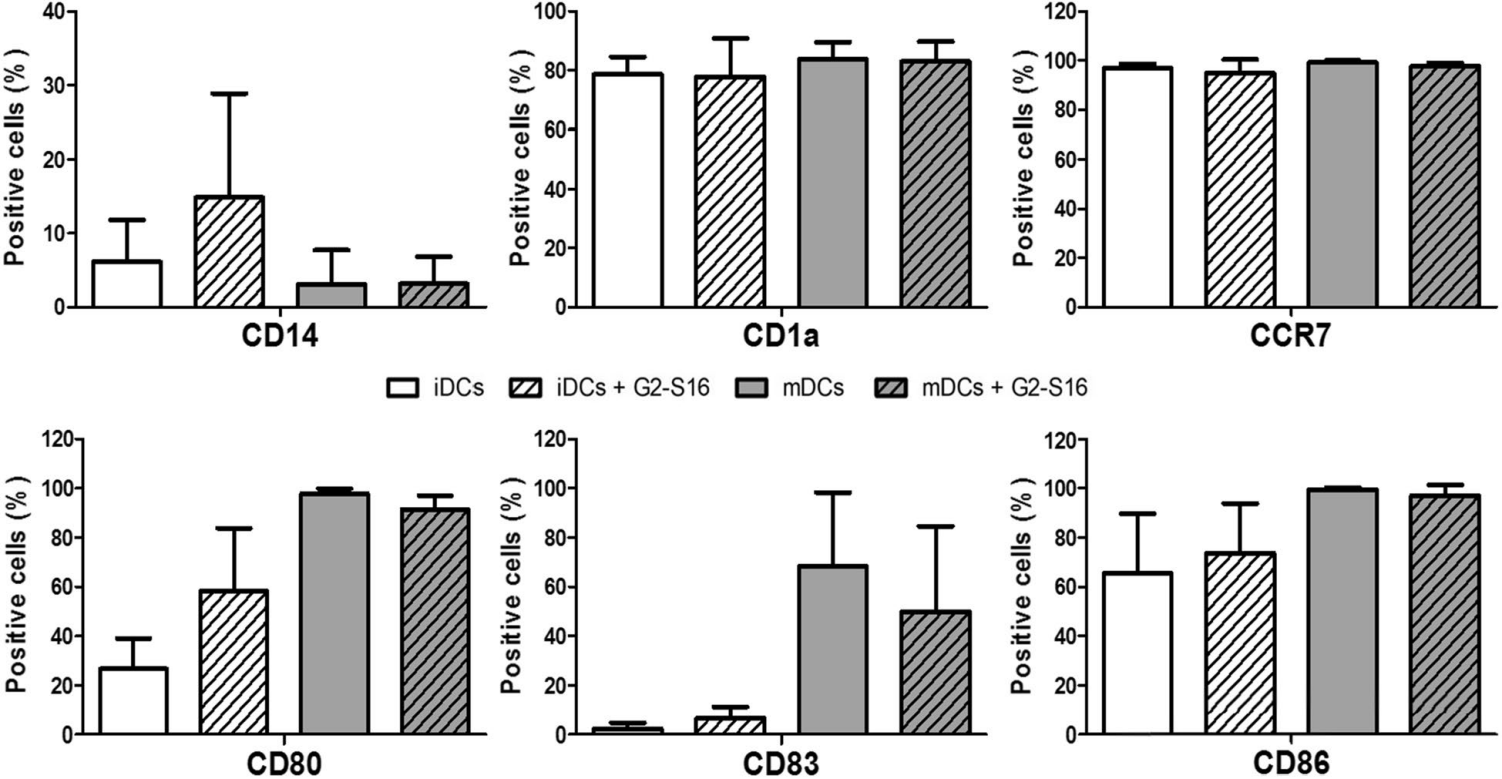
Effect of G2-S16 on maturation of DCs. iDCs were treated with G2-S16 with and without LPA. After 48 h, cells were stained with fluorescent antibodies against CD14, CD1a, CCR7, CD80, CD83 and CD86. DC marker expression was measured by flow cytometry. No significant difference was found between G2-S16 treated and untreated cells

First, we isolated CD14^+^ monocytes from PBMCs by magnetic-activated cell sorting using CD14 microbeads. The CD14^+^ monocytes were then differentiated into iDCs by cultivation with GM-CSF and IL-4 for 5 days. To produce mDCs, iDCs were treated with LPS for 2 days, while the iDCs were maintained by addition of fresh GM-CSF and IL-4. G2-S16 was added at the same time and kept for 48 h during maturation. We then compared the expression of surface markers CD14, CD1a, CD80, CD83 and CD86 in iDCs or mDCs. G2-S16 did not affect the expression of CD14 and CD1a, a type 1 CD1 membrane protein widely used as human DCs markers expressed early in their development (Fig. 5) [27]. As shown in the Fig. 5, iDCs exhibited a similar immature phenotype when treated with G2-S16 compared to non-treated iDCs, although CD80 surface marker showed slight increase, but these results were not significant. Addition of LPS to iDCs induced high levels of expression of co-stimulatory molecules CD83, CD80 and CD86, comparable to what has been published in previous studies [28]. In the presence of G2-S16, DCs treated with LPS also presented a significant increase on maturation markers mDCs (CD80, CD83 and CD86), although slightly lower compared to non-treated mDCs (Fig. 5). Summarizing, G2-S16 did not induce significant changes in CD83, CD80 and CD86 expression levels in iDCs or mDCs, which suggests that the increased expression in TLR2 and TLR7 does not affect to the DC function.

Migration of DCs from the site of HIV capture to the secondary lymphoid organs where they can act in association with T-cells is crucial for initiation of primary immune responses. DCs maturation and migration is correlated with an up-regulation of the chemokine receptor CCR7, in such a way that a decrease of CCR7 and its ligands leads to impaired DC migration into lymph nodes and lymphatic architectural abnormalities in peripheral tissues [29]. Therefore, we evaluated whether migration of mDCs could be altered in the presence of G2-S16. iDCs were obtained as previously explained and were then treated with G2-S16 (1 µM) during maturation. Migration of DCs was evaluated by the expression of surface marker CCR7. The mDCs treated with G2-S16 have a similar ability to migrate compared to the migratory ability of non-treated mDCs (Fig. 5).

### G2-S16 dendrimer does not impede the differentiation from monocytes to M-MØs and GM-MØs and does not affect the phenotype of MØs

MØs are tissue resident phagocytic cells that, together with DCs, constitute the first line of immune cells in the vagina. MØs can be divided in two populations, pro-inflammatory and microbicidal (GM-MØs) or immunosuppressant and tissue repairers (M-MØs). This populations are specially interesting during HIV pathogenesis as the virus takes advantage of their interchangeable polarization [30]. GM and M-MØs have mechanisms to block HIV-1 replication at different steps; GM-MØs inhibit genome integration, while M-MØs inhibit replication at a post-integration stage. HIV-1 fights this by transforming M-MØs into a GM-MØ-like phenotype, thus being able to complete the replication cycle in MØs [31], and blocking this change could result in an effective prophylactic treatment. However, it must be kept in mind that an unbalanced polarization is negative to the host due to tissue damage. Therefore, we evaluated whether G2-S16 affects the differentiation from monocytes to M-MØs and GM-MØs or whether G2-S16 could cause any change in phenotype, thus altering the physiological balance.

**Fig. 6:**
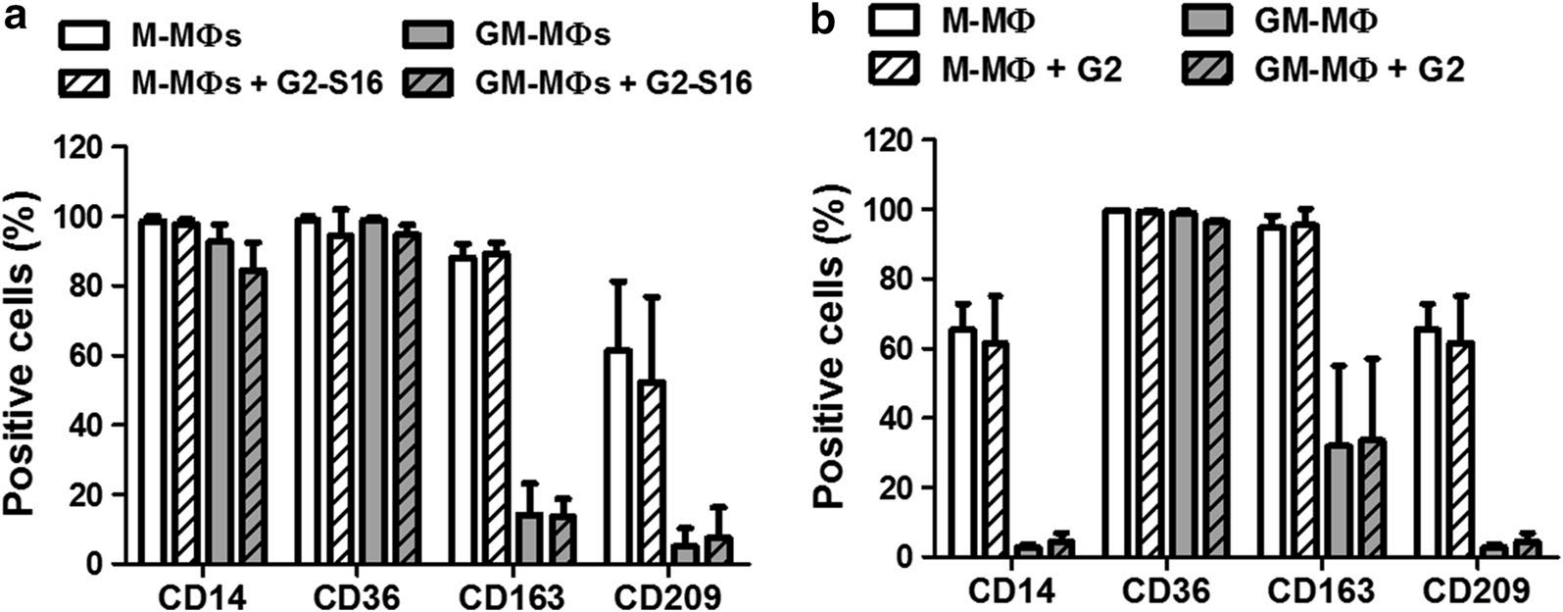
Effect of G2-S16 on the differentiation from monocytes to macrophages and on the phenotype of differentiated M-MØs and GM-MØs. MØs were differentiated from peripheral blood monocytes into M and GM-MØs. The cells were treated with G2-S16 (10 µM) (**a**) during the differentiation process or (**b**) for 48 h after differentiation. MØs marker expression (CD14, CD36, CD163, CD209) was measured by flow cytometry. No significant difference was found between G2-S16 treated and untreated cells

First, we isolated CD14^+^ monocytes from PBMCs by magnetic-activated cell sorting using CD14 microbeads. The CD14^+^ monocytes were then differentiated into MØs by incubation with either GM-CSF or M-CSF for 7 days in the presence of G2-S16 (10 µM) to evaluate the effect of the dendrimer on MØ differentiation. CD36 is a multi-ligand scavenger receptor related to phagocytosis which has been used to identify monocytes and MØs [32]. CD163 and CD209 (also known as DC-SIGN) are highly expressed by M-MØs and in a lesser extent on GM-MØs [33, 34]. Fluorescence-activated cell sorting (FACS) analysis showed that G2-S16 did not modified he expression of monocyte surface markers and did not block the increased expression of the MØ markers, CD36, CD163 and CD209 (Fig. 6a), suggesting that G2-S16 does not affect the monocyte-MØ differentiation. These results are consistent with the fact that G2-S16 does not modify the expression of TLRs.

Once differentiated the MØs in the tissue, they can switch their phenotype between GM-MØs and M-MØs due to interaction with HIV-1 or other STIs pathogens. Therefore, we differentiated MØs according to the above-mentioned protocol and evaluated whether G2-S16 could cause any changes or alter the physiological balance (Fig. 6b). We treated both lineages with G2-S16 for 48 h. FACS analysis showed that G2-S16 did not alter the expression of CD14, CD36, CD163 or CD209 in GM-MØs or M-MØs, suggesting that G2-S16 is safe to respect the MØ balance.

### G2-S16 dendrimer does not modify the populations of CD4^+^ and CD8^+^ T cells or their expression of CCR5 and CXCR4

**Fig. 7:**
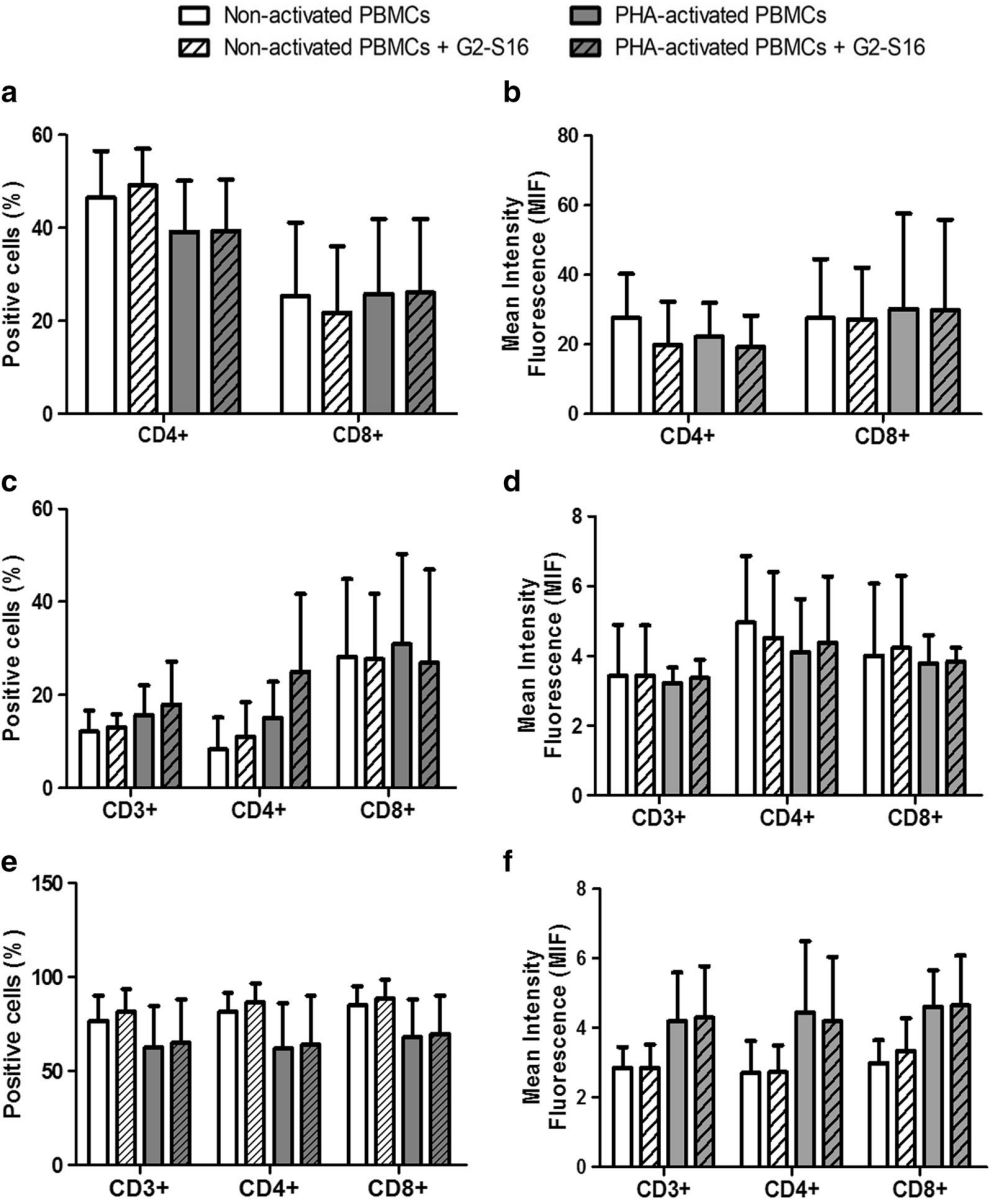
Effect of G2-S16 on the populations of CD4^+^ and CD8^+^ T cells or their expression of CCR5 and CXCR4. Non- and PHA-activated PBMCs were treated with G2-S16 (10 µM) for 48 h and protein expression was determined by flow cytometry. We studied the CD4 and CD8 T cell populations (**a**, **b**), and the expression in these populations of the HIV-1 coreceptors CCR5 (**c**, **d**) and CXCR4 (**e**, **f**). For each marker, the percentage of positive cells (**a**, **c** and **e**) and the mean fluorescence intensity (**b**, **d** and **f**) were analyzed

Lymphocytes are part of the adaptive immune cells, and although they are cells of the adaptive immune system and are not usually considered part of the first line of defense, they are also infiltrated in the FRT, and detect pathogens that get in the tissue, including HIV-1, early in the process of infection. We studied whether G2-S16 modifies the populations of CD4^+^ and CD8^+^ T cells. We had previously shown that G2-S16 (10 µM) inhibits HIV-1 infection in PBMCs, and now we demonstrate that this protective effect is not due to an alteration on the CD4 receptor. PHA-activated or non-activated PBMCs were treated with G2-S16 (10 µM) for 48 h and then the surface proteins on CD3^+^ lymphocytes were measured by flow cytometry. No significant differences were observed in the binding of the anti-CD4 and anti-CD8 antibodies to the CD4 and CD8 cellular receptors. These results show that G2-S16 does not modify the percentage of CD4 or CD8 positive T cells, neither in basal state nor in PHA-activated PBMCs (Fig. 7a). A comparison of the mean fluorescence intensity (MFI) was used to verify that the amount of antibody bound, and thus, of protein expressed per cell was also not altered by G2-S16 dendrimer (Fig. 7b).

We also studied the expression of the HIV-1 co-receptors CCR5 and CXCR4 in the G2-S16 treated PBMCs. The experiment was performed in the same conditions, but antibodies against CCR5 and CXCR4 were used for flow cytometry. No significant difference was observed on the number of cells expressing the co-receptors after exposure to the G2-S16 dendrimer (Fig. 7c, e). The amount of protein exposed in the cell surface was also not altered after exposure to G2-S16, as shown by the comparison of the (MFI) (Fig. 7d, f).

### Toll-like receptor expression in CD4-T cells remains unaltered in the presence of G2-S16 dendrimer

**Fig. 8:**
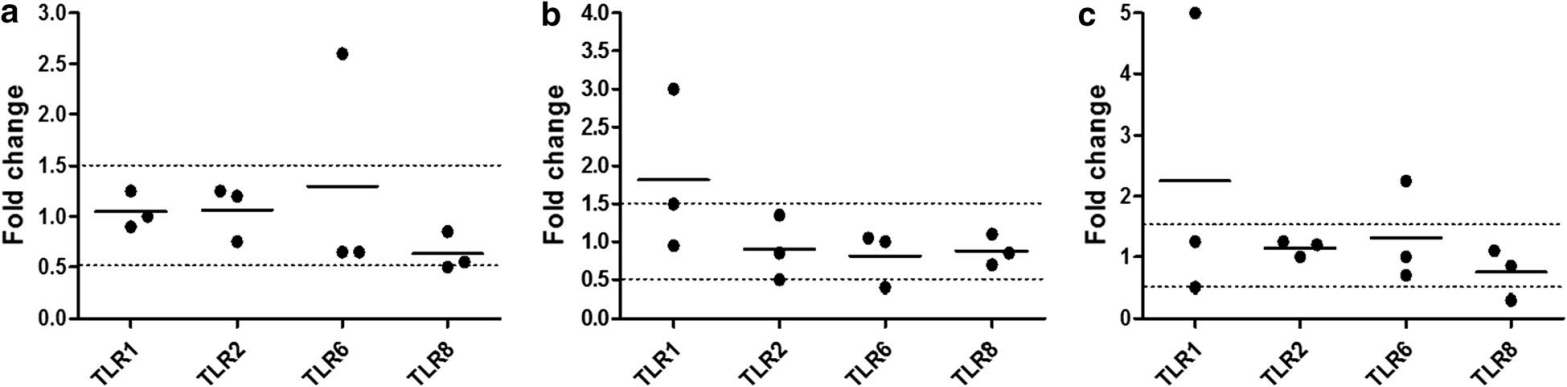
Quantification of gene expression on treated CD4-T cells by real time RT-qPCR. TLR1–10 mRNA expression was determined by RT-qPCR in CD4-T cells treated with G2-S16 (10 µM) for **a** 3 h, **b** 6 h and **c** 18 h. Insufficient detection of certain TLR mRNA expression levels by RT-PCR was not included here to simplify the representation. Data shows relative mRNA levels; analyzed gene was normalized to TATA box binding protein expression (TBP) and referred to non-treated cells. Individual values (dots) and mean (bar) of at least three independent experiments are shown. Dashed lines indicate threshold values

Although TLRs are primarily believed to dictate the innate immune response, increasing data is providing evidence of role of the expression and activation of TLRs in T cells, and thus, the adaptive immune response [35, 36]. We evaluated the expression of TLR1–10 in CD4^+^ T cells after treatment with G2-S16 (10 µM) for short (3 h), medium (6 h) or long (18 h) times (Fig. 8). We used the forward and reverse primer pairs described in Table 1, and TLR1, 2, 6 and 8 were expressed at a detectable amount. TLR1 showed a slight increase (over 1.5 folds) at 6 h (Fig. 8b) and 18 h (Fig. 8c), but the difference was not statistically significant. Altogether, these data suggest that G2-S16 dendrimer does not have a significant effect on the expression of TLRs in CD4^+^ T cells at the time points studied.

### G2-S16 dendrimer does not modify the activation state of T-cells

**Fig. 9:**
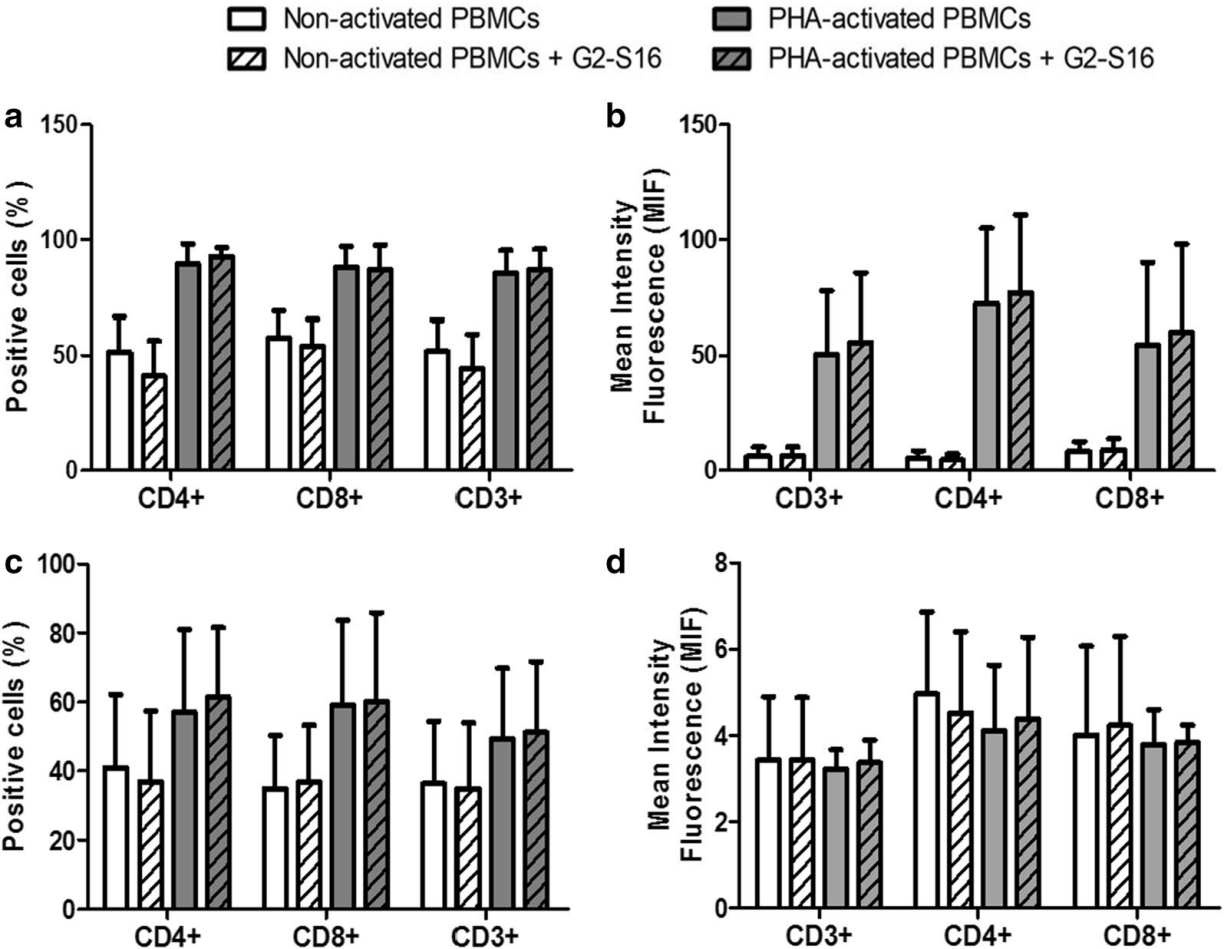
Effect of G2-S16 on the activation state of T-cells. Non- and PHA-activated PBMCs were treated with G2-S16 (10 µM) for 48 h and protein expression was determined by flow cytometry. We studied the activation markers for early activation, CD69 (**a**, **b**) and late activation HLA-DR (**c**, **d**). For each marker, the percentage of positive cells (**a**, **c**) and the mean fluorescence intensity (**b**, **d**) were analyzed

The previous results altogether suggest that G2-S16 does not activate the immune system, as it does not cause any maturation changes in the APCs and does not activate T cells via TLR signaling either. To further prove that the adaptive immune cells are not altered by the dendrimer, we evaluated the expression of activation markers in PBMCs. Upon T-cell activation, several cell surface markers are up-regulated at different stages of the activation process. We chose CD69 are early activation marker, and HLA-DR as late activation marker [37]. PBMCs were treated with G2-S16 (10 µM) for 48 h and the levels of surface markers on CD3^+^ lymphocytes were then measured by flow cytometry. No significant changes were observed on activation markers after treatment with G2-S16 compared to non-treated controls (Fig. 9) in CD3^+^CD4^+^ or CD3^+^CD8^+^ cells. Consistently with the previous assays, these results indicated that G2-S16 dendrimer does not affect the activation of the immune cells in the absence of an exogenous antigen.

### G2-S16 does not modify the population or activation state of B cells

**Fig. 10:**
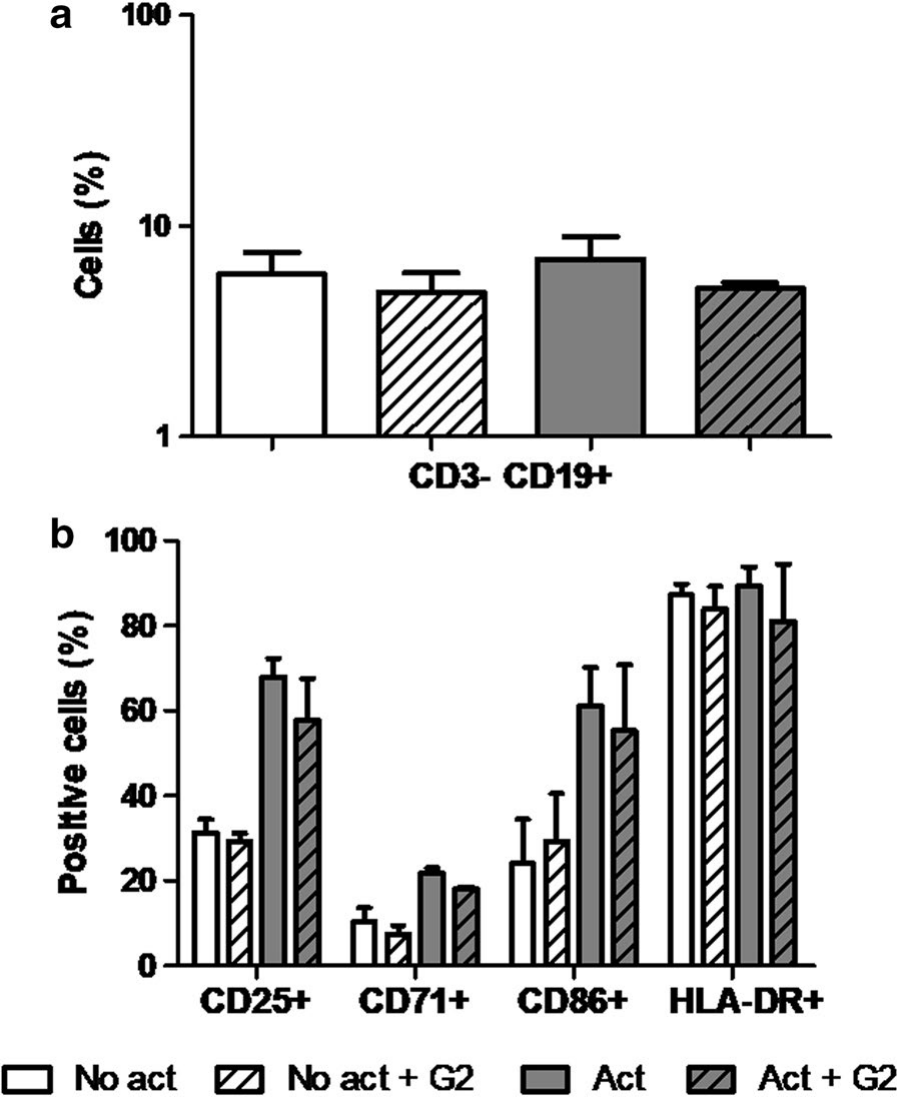
Effect of G2-S16 on B cell population and B cell activation. Not activated and PBMCs activated with IL-4 and CD40L were treated with G2-S16 (10 µM) for 48 h and protein expression was determined by flow cytometry and the percentage of positive cells was analyzed for each marker. **a** The B cell population was identified as CD3^−^ CD19^+^ cells. **b** We studied the standard activation markers for the B cell population: CD25, CD71, CD86 and HLA-DR

B cells are the lymphocytes responsible for the mucosal antibody defense. In order to evaluate whether G2-S16 affects the B cell population, we treated PBMCs with G2-S16 (10 µM) for 48 h and measured the B cell population by flow cytometry as CD3^−^ CD19^+^ cells, both in non-activated and IL-4-CD40L activated PBMCs. We observed no significant difference in the percentage of B cells with or without treatment with the dendrimer (Fig. 10a).

We also studied the activation of the B cells under the same conditions. With that objective, we measured the expression of B cell activation markers CD25, CD71, CD86 and HLA-DR by flow cytometry, and found that G2-S16 does not modify the expression of activation markers (Fig. 10b), thus suggesting that it does not affect B cell function.

In summary, the results presented in this study demonstrate that G2-S16 dendrimer does not modify the immune barrier of the FRT, providing stronger evidence of its safety to go to clinical trials as prophylactic treatment against HIV-1 infection.

## Discussion

Preventive topical microbicides against HIV-1 infection are designed to be applied in the vagina or rectum and remain there before, during and after sexual intercourse. In the case of HIV-1 exposure, the microbicide should prevent the infection, but it should not interfere with the normal function of the local immune system. This last fact is key for the safety of the compound, but it is frequently overlooked. Many compounds aimed to serve as microbicides fail in clinical trials due to unexpected toxicities or side effects. The objective of this study is to prove the safety of the potential HIV-specific microbicide G2-S16 dendrimer with regards to the vaginal and rectal immunity.

G2-S16 is a second generation of anionic carboxilane dendrimer that has been shown to prevent HIV-1 infection in vitro [19] and in vivo using humanized BLT mice [38]. G2-S16 has been proven to block HIV infection in multiple cell lines and human primary blood cells Chonco et al. [19]. This dendrimer inhibits infection even in the presence of semen, a known infection enhancer [17, 39], as well as the cell-to-cell transmission and syncytium formation [40]. On the other hand, the biosafety of this nanocompound has been tested not only in vitro but also ex vivo and in vivo [19, 20, 38]. Cell viability after exposure to G2-S16 remained unaltered for several cell lines and primary cells, including lymphocytes, DCs and MØs. The biocompatibility was also tested in EpiVaginalTM human vaginal epithelial tissue, by MTT assay [20]. Finally, animal models including mice and rabbits were used to assess and confirm the safety of G2-S16 regarding vaginal epithelium irritation and inflammation [20]. These results altogether present G2-S16 as a promising candidate for topical microbicide against HIV-1 infection, situating this dendrimer on the way to clinical trials.

The results presented in our study show that G2-S16 is not only efficient in HIV-1 prevention and safe regarding cell survival, but it also respects the phenotype of the main immune cells exposed. The sexual transmission of the HI-1 can occur through the vagina or the rectus, and the main immune cells exposed to this microbicide will be the innate immune cells in the vaginal mucosa, DCs and MØs, and the lymphocytes after epithelial damage in the rectum. DCs and MØs are tissue resident cells that differentiate from monocytes. Our study demonstrates that the exposure to G2-S16 does not modify the differentiation of monocytes to either DCs or MØs. DCs patrol the mucosal tissue in the search for pathogens, and upon detection of an antigen, they undergo maturation and migrate towards the lymph nodes, where they activate naïve lymphocytes. DC maturation implies the expression of membrane proteins responsible for cell migration (CCR7) or T cell activation (CD80, CD83 and CD86). Our results show that the exposure of differentiated DCs to the dendrimer does not modify the levels of maturation achieved after exposure to an antigen, such as LPS. MØs are divided into two main populations known as M and GM-MØs, which work as tissue-repair cells and pro-inflammatory cells, respectively. Under specific circumstances, the populations can be modified to create a pro-inflammatory or anti-inflammatory environment. However, in order to maintain tissue homeostasis, the equilibrium between both populations is key and any alteration caused by an external treatment, such a prophylactic drug, could have unpredicted and highly damaging consequences. Our results show that MØ-populations exposed to G2-S16 do not suffer any alteration in the ratio of M/GM-MØs.

Differently from what happens in the vagina, in the case of rectal transmission of HIV-1, the first cells infected are the CD4^+^ lymphocytes from the bloodstream, after the virus crosses the epithelium through micro wounds that occur during sexual intercourse. The prophylactic treatment would be applied in the rectum and similar to what happens with the virus, it would get in touch with the immune cells in the bloodstream. Our results show that G2-S16 does not modify the percentage of CD4 or CD8 positive cells, neither in basal state not in PHA-activated PBMCs. In each population, G2-S16 also does not alter the amount of antibody bound, and thus, the amount of protein expressed per cell. These results also discard the option of G2-S16 binding to CD4 as a mechanism of protection against HIV-1, as the same amount of antibody was able to bind to the treated cells compared to the untreated, thus suggesting that G2-S16 does not block the binding to the virus either. Under the same experimental conditions, these results also showed that the expression of HIV-1 co-receptors CCR5 and CXCR4 remained unchanged after exposure to G2-S16, both when looking at the percentage of cells or the protein expression. Although TLRs are primarily believed to dictate the innate immune response, increasing data is providing evidence of the role of the expression and activation of TLRs in T cells, and thus, the adaptive immune response [35, 36]. Our data clearly show that G2-S16 does not have a significant effect on the expression of TLRs in CD4^+^ T cells at the time points studied.

Up until this point, our results suggest that G2-S16 does not activate the immune system, as it does not cause any maturation changes in the APCs and does not activate T cells via TLR signaling either. The evaluation of the expression of the T-cell maturation markers, CD69 and HLA-DR for early and late activation respectively [37], further proved that G2-S16 does not affect the activation of the immune cells in the absence of an exogenous antigen.

These results also discard the option of G2-S16 binding to CD4 as a mechanism of protection against HIV-1, as the same amount of antibody was able to bind to the treated cells compared to the untreated, thus supporting the hypothesis that it binds to the viral surface. G2-S16 dendrimer has been demonstrated to protect against HIV-1 infection when used as prophylactic treatment both in vitro and in vivo, but the mechanism of this protection remains controversial. Computational modeling assays show that it disrupts the union of the virus to the host cell, but they show that it could bind to residues in both the viral gp120 and the CD4 cellular receptor [40]. The cytometry assays performed here show that G2-S16 does not block the binding of antibodies to CD4 receptor, and thus suggest that it does not block the binding of the virus either.

We studied the most likely facts that could be affected by the dendrimer, but other membrane proteins or factors could be altered. Also, it could affect other cell types present in the FRT, including NKs or B cells. Although it is unlikely that the dendrimer could affect these cell types, and we analyzed here the main ways the dendrimer could alter and harm the normal immune function, further studies in this field would be useful to totally grant the non-interaction of G2-S16 dendrimer with the IS.

## Conclusions

Summarizing, in this study we evaluated the effect of G2-S16 dendrimer on the epithelial and immune cells present in the FRT with the aim of identifying potential negative effects prior to clinical trials. These data provide a widen understanding and a more rigorous preclinical tool for the safety evaluation of G2-S16. Our in vitro findings show that G2-S16 dendrimer can slightly interfere with TLR responses on different cell populations, mostly on DCs, but the over-expression of the receptor does not affect the cell functions or the expression of other relevant markers. Epithelial cells, DCs, MØs and lymphocytes, which are the main components of the first barrier of defense in the FRT and rectus are not significantly altered by G2-S16, providing stronger evidence that the dendrimer is safe for clinical use. Further in vitro and in vivo studies would be useful to validate these in vitro results. Additional in vitro assays should include effects on viability of B-cells, target cell recognition by natural killer cells and cell trafficking from the vaginal mucosa.

## Materials and methods

### Reagents

Water-soluble polyanionic carbosilane dendrimer G2-S16 (C_112_H_244_N_8_Na_16_O_48_S_16_Si_13_; Mw = 3717.15 g/mol) was synthesized according to methods reported by the University of Alcalá [41]. G2-S16 consists of a second-generation dendrimer scaffold built from a silicon atom core and fully capped on the surface with 16 sulfonate groups. The second-generation was defined taking into account the number of repeated layers of silicon atoms forming the dendrimer. A 10 mM stock solution of G2-S16 and subsequent dilutions to obtain µM concentrations were prepared in nuclease-free water (Promega, Madison, WI, USA).

### Cell line culture

Vaginal epithelium VK2/E6E7 cells (ATCC^®^ CRL-2616™, Manassas, VA, USA) were seeded at 0.25 × 10^6^ cells/mL and cultured in keratinocyte serum-free medium (Gibco, Paisley, UK) with 0.1 ng/mL human recombinant epidermal growth factor (hrEGF; Immunotools, Friesoythe, Germany), 0.05 mg/mL bovine pituitary extract (BPE; Sigma-Aldrich, St. Louis, MO, USA) and calcium chloride (Sigma) at 0.4 mM.

### Primary cell cultures, purification and differentiation

Human peripheral blood mononuclear cells (PBMCs) were isolated from buffy coats obtained from anonymized healthy blood donors coming from the transfusion Center of Madrid following national guidelines. PBMCs were isolated by a standard *Ficoll*-*Hypaque* density gradient (Rafer, Madrid, Spain) and cultured following the procedures of Spanish HIV HGM BioBank [42–45]. PBMCs were kept with 60 U/mL of interleukin 2 (IL-2; Bachem AG, Bubendorf, Switzerland) and stimulated with 60 U/mL of interleukin 2 (IL-2; Bachem AG, Bubendorf, Switzerland) and 2 μg/mL of phytohemaglutinin (PHA; Remel, Santa Fe, NM, USA) for 48 h before the experiments for T cell analysis. For B cell studies, PBMCs were stimulated with 50 ng/mL interleukin 4 (rhIL-4; Immunotools) and 500 ng/mL CD40 ligand (CD40L, Invitrogen).

CD4-T cells were purified from PBMCs using immunomagnetic anti-CD4 microbeads (CD4 MicroBeads; Miltenyi Biotec, Bergisch Gladbach, Germany). Purified CD4-T cells were seeded at 5 × 10^6^ cells/mL in RPMI-1640 medium (Biochrom AG, Berlin, Germany) with 10% fetal bovine serum (FBS; Gibco), 1% l-glutamine (Lonza, Walkersville, MD, USA), antibiotic cocktail (125 mg/mL ampicillin, 125 mg/mL cloxacillin and 40 mg/mL gentamicin; all from Normon, Madrid, Spain) and 60 U/mL of IL-2.

Monocytes were purified using immune-magnetic anti-CD14 microbeads (Miltenyi) and were seeded under different conditions depending on the cell type to which would differentiate.

Immature DCs (iDCs) and mature DCs (mDCs): Monocytes were seeded at 10^6^ cells/mL in RPMI-1640 medium supplemented with 10% FBS, 1% l-glutamine, 50 μM β-2-mercaptoethanol (Sigma), 20 ng/mL recombinant human interleukin 4 (rhIL-4; Immunotools) and 50 ng/mL recombinant human granulocyte macrophage colony stimulating factor (rh GM-CSF; Immunotools), and were maintained during 5 days. Culture medium was renewed the 3rd day. iDCs were cultured for 48 h in the presence of lipopolysaccharide (LPS; Sigma) at 20 ng/mL to stimulate maturation.

GM-MØs: Monocytes were cultured at 0.5 × 10^6^ cells/mL for 7 days in RPMI-1640 medium supplemented with 10% FBS; 1% l-glutamine and 10 ng/mL of rhGM-CSF to generate GM-MØs. Cytokines were added every 2 days.

M-MØs: Monocytes were cultured at 0.5 × 10^6^ cells/mL for 7 days in RPMI-1640 medium supplemented with 10% FBS, 1% ʟ-glutamine and 10 ng/mL of recombinant human macrophage-colony stimulating factor (rhM-CSF; Immunotools) to generate M-MØs. Cytokines were added every 2 days.

### Cell viability assay

The tetrazolium dye colorimetric assay is a method for testing cell cytotoxic activity in vitro. The 3-(4,5-dimethylthiazol-2-yl)-2,5-diphenyl-tetrazolium-bromide (MTT; Sigma) was used following the manufacturer’s instructions to determine the viability of DCs and MØs (cell density of 7.5 × 10^4^ and 5 × 10^4^ cells/mL, respectively) in the presence of G2-S16 dendrimer or controls.

### Flow cytometry

Analysis of cell-surface phenotype of DCs and MØs was performed by flow cytometry. Cells were labeled with anti-CD4-FITC, anti-CD69-PC5, anti-CD80-FITC, anti-CD1a-PE, anti-HLA-DR-ECD, anti-CD14-PC7, anti-CD86-PC5.5 (all from Beckman Coulter Inc., Brea, CA, USA), anti-CD8-Pacific Blue, anti-CD36-APC, anti-CD197-APC-Cy7 (CCR7) (all from BioLegend, San Diego, CA, USA), anti-CCR5-PE, anti-CD209-PE, anti-CD83-APC (all from BD Biosciences, Franklin Lake, NJ, USA), anti-CXCR4-APC (RandD systems, Minneapolis, MN, USA) and anti-CD163-FITC (MBL International Corp., Woburn, WA, USA). Levels of surface expression on cells were estimated by flow cytometry (Gallios; Beckman) and analyzed using Kaluza software (Beckman).

### Treatment of cell cultures for RNA extraction

After cells were purified and/or differentiated, and seeded at appropriate conditions (indicated above), were treated with G2-S16 at the highest non-toxic dose considering the cell line evaluated in this study or previously reported [18, 19]. In adherent culture cells, after 3 h, 6 h and 18 h of treatment, the supernatants were removed and cells were washed once with phosphate-buffered saline (PBS; Lonza), then the RNA extraction was performed. In suspension cultures, after 3 h, 6 h and 18 h of treatment, the cells were placed in a tube and centrifuged at 600×*g* for 10 min, supernatants were removed and pellet were washed once with PBS, then, centrifuged at 600×*g* for 10 min and the pellet was used to perform the cellular RNA extraction.

### RNA extraction and TLR mRNA expression detection by RT-qPCR

RNA of all samples (adherent cells or pellet cells) was extracted using the RNeasy Plus mini kit (Qiagen, Valencia, CA, USA) and RNA integrity was analyzed with Agilent 2100 bioanalyzer (Agilent Technologies, Palo Alto, CA, USA) by using RNA Nano chips (Agilent). To perform the process of reverse transcription, a reverse transcriptase was used to generate complementary DNA (cDNA) from RNA templates with the GoScript Reverse Transcription System (Promega). mRNA expression was analyzed by quantitative real-time PCR using specific probes and primers (see Table 1) for TLR1, TLR2, TLR3, TLR4, TLR5, TLR6, TLR7, TLR8, TLR9 and TLR10 designed by using the Universal Human Probe Roche library system (Roche Diagnostics, Basel, Switzerland). Results were processed with the Bio-Rad iQ5 2.0 software (Hercules, CA, USA). Data was normalized according to the expression levels of TATA-binding protein (TBP) mRNA, used as a housekeeping gene, and expressed the fold change relative to the mRNA level of untreated samples (value = 1). The 1.5 and 0.5 folds change were established as threshold values.

## Data Availability

All the reagents will be made available to other researcher upon previous request.
